# Metabologenomic Approach Reveals Intestinal Environmental Features Associated with Barley-Induced Glucose Tolerance Improvements in Japanese: A Randomized Controlled Trial

**DOI:** 10.3390/nu14173468

**Published:** 2022-08-24

**Authors:** Yuka Goto, Yuichiro Nishimoto, Shinnosuke Murakami, Tatsuhiro Nomaguchi, Yuka Mori, Masaki Ito, Ryohei Nakaguro, Toru Kudo, Tsubasa Matsuoka, Takuji Yamada, Toshiki Kobayashi, Shinji Fukuda

**Affiliations:** 1 Hakubaku Co., Ltd., Chuo, Yamanashi 409-3843, Japan; 2 Metagen Inc., Tsuruoka, Yamagata 997-0052, Japan; 3 Institute for Advanced Biosciences, Keio University, Tsuruoka, Yamagata 997-0052, Japan; 4 Department of Life Science and Technology, Tokyo Institute of Technology, Meguro, Tokyo 152-8550, Japan; 5 Gut Environmental Design Group, Kanagawa Institute of Industrial Science and Technology, Kawasaki-ku, Kawasaki, Kanagawa 210-0821, Japan; 6 Transborder Medical Research Center, University of Tsukuba, Tsukuba, Ibaraki 305-8575, Japan

**Keywords:** gut microbiome, intestinal metabolome, prebiotics, dietary fiber, barley

## Abstract

(1) Background: Consumption of barley has been known to exert beneficial effects on glucose tolerance; however, it has also been reported that there are inter-individual differences in these responses. Recent evidence has suggested that these individual differences are mediated by the gut microbiota. (2) Methods: In the present study, we aimed to understand the relationship between the intestinal environment, including intestinal microbiome and their metabolome, and glucose tolerance. A randomized controlled trial with a 4-week consumption of barley or control food was conducted. We conducted an integrated analysis of the intestinal microbiome and metabolome and analyzed the relationship with improvement of glucose tolerance. (3) Results: We found that metabolites such as azelate were significantly increased after barley consumption. Furthermore, the subjects whose glucose tolerance was slightly impaired showed improvement in their glucose tolerance index following the barley consumption. Additionally, the analysis showed that the increase in the abundance of the *Anaerostipes* was correlated with the improvement in the glucose tolerance index. (4) Conclusions: Our findings indicate that the effects of barley consumption for glucose tolerance are partly defined by the intestinal environment of consumers, providing a quantitative measurement of the dietary effect based on the intestinal environment.

## 1. Introduction

Barley was one of the first domesticated grains and has been cultivated for about 10,000 years. Recent studies have revealed that barley has the potential to improve certain blood parameters, such as glucose tolerance and total blood cholesterol [1,2,3]. As barley contains a high amount of β-glucan, a type of soluble fiber, its effect on these blood parameters have been attributed to its high viscosity. It has been reported that barley-derived β-glucan conjugates with sugars and lipids in the human gastrointestinal lumen to inhibit their digestion and absorption [4,5]. In addition, a previous study showed that barley consumption increases the *Prevotella*/*Bacteroides* ratio in barley-consuming healthy Swedish subjects with improved glucose tolerance [6]. These reports indicate that β-glucan affects blood glucose levels in two ways: directly by binding to sugars, and indirectly through changes in the intestinal environment.

Despite these studies, the underlying relationship between barley intake and glucose intolerance is yet to be revealed, as many reports suggest that there are multiple confounding factors. First, genetic background affects the blood parameters. It has been reported that the primary symptom in the early stage of diabetes is different in populations from different backgrounds. For example, Caucasians show insulin resistance, whereas East Asians show a decrease in insulin secretion [7]. Second, it has been suggested that the effect of barley is partly due to the functions of the intestinal microbiome [6]; however, within the same species of bacteria, the existence of different strains unique to each country has been implied. It has also been proposed that the differences in these strains can possibly lead to differences in the phenotypes. For instance, various strains of Prevotella copri have been observed in multiple countries, and their functions are dissimilar [8]. Moreover, there have been no studies reporting the effect of barley on the intestinal environment and glucose tolerance index in Asian populations, such as the Japanese, who are known to have unique microbiomes [9].

As explained above, the effect of barley intake on the intestinal microbiome and metabolome profiles is still unclear. To elucidate the mechanisms by which barley improves the blood parameters through the intestinal environment in Japanese subjects, a randomized double-blind controlled trial was conducted. The intestinal environment was evaluated using a metabologenomic approach, which combines mass spectrometry-based metabolome and high-throughput sequencing-based microbiome analyses.

## 2. Materials and Methods

### 2.1. Ethics Approval

The human rights of the subjects who participated in this study were protected at all times, and the study observed the Helsinki Declaration and Ethical Guidelines on Epidemiological Research in Japan referring to cases concerning standards for clinical trials of drugs. Informed consent was obtained from all individual participants and preserved in the text. This trial was conducted with the approval of the clinical trial ethics review committee of Chiyoda Paramedical Care Clinic (publicly registered at University hospital Medical Information Network Clinical Trials Registry, Trial number: UMIN000023675).

### 2.2. Trial Design and Recruitment

In this study, a randomized double-blind controlled trial with Japanese participants was performed for 3 months (Table S1). The study included 4-week dietary intervention periods (Period 1 and 2), comprising a test food (22 g barley with 138 g multi grain rice) and control food (barley-free 150 g multi grain rice) in random order and separated by a 4-week washout period (Washout). The calories in the test food and control food were standardized in the trial although their weights were different; test food and control food calories were 465 kcal and 468 kcal, respectively. Barley was shaped by cutting and pearling to mimic rice. During the dietary intervention periods, subjects were instructed to substitute the staple food in their diet with the test or control food twice a day. The test food and control food were preliminarily processed to prevent subjects from identifying the difference visually. The trial was initiated in July 2016 and completed in December 2016.

During the trial, fecal samples were collected at baseline (T1 and C1, T = test food and C = control food), 2 weeks (T2 and C2), and 4 weeks (T3 and C3) of the dietary intervention periods. The collected stool samples were frozen at −20˚C until processing. In addition, clinical blood tests and oral glucose tolerance tests were performed following a 12-h fasting at the same time points. In the clinical blood test, total protein, albumin, aspartate aminotransferase, alanine transaminase, lactate dehydrogenase, total bilirubin, alkaline phosphatase, γ-glutamyl transpeptidase, creatine phosphate enzyme, urea nitrogen, creatinine, uric acid, sodium, chlorine, potassium, calcium, total cholesterol, low-density lipoprotein cholesterol, high-density lipoprotein cholesterol, neutral fat, glucose, insulin, Hemoglobin A1c, white blood cell, red blood cell, hemoglobin, hematocrit, and platelet were measured. For the oral glucose tolerance test, blood samples were taken at 30, 60, 90, and 120 min after the oral administration of 75 g glucose for glucose and insulin level measurements. The area under the curve (AUC) and incremental AUC (iAUC) were calculated using the trapezoid model.

In total, 48 participants were recruited for pre-trial and 24 participants were enrolled in this study. The enrolled participants fulfilled the following criteria: aged between 50 and 69 years old, body mass index of 18–25 kg/cm^2^, and fasting plasma glucose level under 109 mg/dL. Detailed inclusion/exclusion criteria are shown in Table S2. Prior to the main trial, the blood tests were conducted. Based on the preliminary blood test and male-female ratio, 24 subjects were selected for the main trial. All 24 subjects completed the trial; however, five subjects were excluded from the analysis for following reasons: incomplete blood sampling (subject 11), incomplete fecal sampling (subject 13), and consumption of prohibited food such as fermented products during the trial (subjects 08, 18, and 22). The primary outcome was blood glucose and insulin AUC after 2 weeks and 4 weeks of test food consumption. In addition, the key secondary outcome was blood glucose and insulin iAUC, fasting blood glucose and insulin, and stool frequency.

### 2.3. Trial Intervention: Randomization and Blinding

Randomization in this trial was performed using the blocked stratified randomization method with the subject assignment manager. First, 24 subjects who passed the inclusion criteria were assigned to two groups (group A and B) by stratification of 12 subjects each, taking into consideration the age and male-female ratio immediately before the trial period. Subsequently, the symbols “A” or “B”, representing the test food and control food, respectively, were randomly assigned to each group of subjects. Following this, a test food assignment table with the test food symbol and the subject identification code was prepared. Immediately after the assignment to the test food according to the assignment table, the table was sealed and kept tightly concealed by the subject assignment manager. The table was disclosed to the test analyst, investigator, and test sharing doctor after data fixation.

### 2.4. DNA Extraction and 16S rRNA Gene-Based Microbiome Analysis

DNA extraction from fecal samples was performed as previously reported [10]. After extraction, the V1-V2 variable region of the 16S rRNA gene was amplified using bacterial universal primers 27F-mod (5′-AGRGTTTGATYMTGGCTCAG-3′) and 338R (5′-TGCTGCCTCCCGTAGGAGT-3′) with Tks Gflex DNA Polymerase (Takara Bio Inc., Otsu, Japan) [11]. The amplicon DNA was sequenced using MiSeq (Illumina, San Diego, CA, USA) according to the manufacturer’s protocol. All 16S rRNA amplicon sequence files generated in this study are available in DRA of DDBJ (DRA accession number: DRA009319).

### 2.5. Metabolite Extraction and CE-TOFMS-Based Metabolome Analysis

Extraction of metabolites from fecal samples was performed as previously reported [12]. Briefly, the samples were initially lyophilized using the VD-800R lyophilizer (TAITEC Co., Ltd., Saitama, Japan) for at least 24 h. Freeze-dried feces were disrupted with 3.0 mm zirconia beads by vigorous shaking (1500 rpm for 10 min) using the Shake Master neo (Biomedical Science Co., Ltd., Tokyo, Japan). Next, 500 μL of methanol, including the internal standards (20 μM each of methionine sulfone and D-camphor-10-sulfonic acid (CSA)), was added to 10 mg of disrupted feces. Samples were further disrupted with 0.1 mm zirconia/silica beads by vigorous shaking (1500 rpm for 5 min), then 200 μL of ultrapure water and 500 μL of chloroform were added before centrifugation at 4600× *g* for 15 min at 20 °C. Subsequently, 150 μL of the aqueous layer was transferred to a centrifugal filter tube (Ultrafree MC-PLHCC 250/pk for Metabolome Analysis, Human Metabolome Technologies, Yamagata, Japan) to remove protein and lipid molecules. The filtrate was concentrated by centrifugation and dissolved in 50 μL of ultrapure water immediately before coupling capillary electrophoresis with electrospray ionization time-of-flight mass spectrometry (CE-TOFMS) analysis. After identifying the peak from CE-TOFMS, relative peak area data, which is a comparison value with internal standards, was obtained. Of these, quantitative values of some metabolites were obtained by comparing with reference material. The obtained metabolome relative peak area and quantitative values are available in Tables S3 and S4, respectively.

### 2.6. Bioinformatics and Statistical Analysis

For 16S rRNA gene analysis, QIIME2 (version 2019.10) was used [13]. In the analytical pipeline, sequence data were processed by using the DADA2 pipeline for quality filtering and denoising (options: --p-trim-left-f 20 --p-trim-left-r 19 --p-trunc-len-f 240 --p-trunc-len-r 140) [14]. The filtered output sequences were assigned to taxa by using the “qiime feature-classifier classify-sklearn” command with the default parameters. Silva SSU Ref Nr 99 (version 132; The SILVA ribosomal RNA database project, Bremen, Germany) was used as a reference database for taxonomy assignment [15]. The microbiome data are available in Table S5.

For the statistical analysis to compare primary and secondary outcomes, we used paired *t*-test and paired Hedge’s g using R package effectsize (R version 3.6.1 and effectsize version 0.4.4.1). All the other statistical analyses described below were performed using Python scripts (version 3.7.3). Multidimensional scaling was performed using unweighted/weighted UniFrac distance and Spearman correlation distance calculated from the data of microbiome OTUs and metabolite relative peak area, respectively (scikit-learn version 0.21.2). In addition, intra-subject-distance was compared with inter-subject distance by PERMANOVA test (scikit-bio version 0.5.5). For the pairwise comparison in blood test data, relative abundance of intestinal bacteria and relative peak area of intestinal metabolite, Wilcoxon signed-rank test with Benjamini–Hochberg false discovery rate (FDR) correction was used (scipy version 1.3.1 and statsmodels version 0.10.1, respectively). During the comparison, bacteria with mean relative abundance below 0.001 and metabolite that was not detected at 75% sample were excluded. In the responder feature analysis, Spearman rank correlation coefficient and test for no correlation was used (scipy version 1.3.1).

### 2.7. Defining Responders with Specific Response

We adopted criteria of glucose tolerance responders reported in the previous study [6], in which responders must fulfill the following criteria: (1) comparing T2 and C2 or T3 and C3, blood glucose iAUC (0–90 min) decreased by at least 25%; (2) comparing T2 and C2 or T3 and C3, blood glucose AUC (0–90 min) decreased; (3) comparing T2 and C2 or T3 and C3, insulin iAUC (0–90 min) decreased by at least 15%. In this study, the test food effect size for each subject was defined as the responder score and used to evaluate whether effects depended on individual basal characteristics. The response score was calculated with the following equation:

Responder Score = ((T3 − T1) − (C3 − C1))/Average (C1, T1)

## 3. Results

### 3.1. The Effect of Barley Intake on Primary and Secondary Outcomes

**Figure 1:**
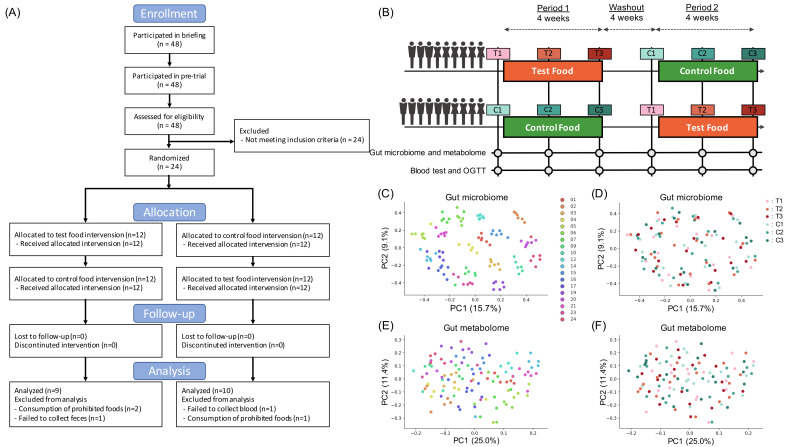
Overview of the double-blind randomized crossover trial (**A**) Flow diagram of this trial. (**B**) Outline figure of this trial. Two 4-week dietary treatments were set in succession. The dietary intervention periods were interspaced by a 4-week washout period. Blood and stool samples were collected before and after 2 and 4 weeks of each intervention period. (**C**,**D**) Scatter plots showing results of multidimensional scaling using beta diversity (unweighted UniFrac distance) calculated from microbiome profile. Plots were color-coded by subject (**C**) or time point (**D**). (**E**,**F**) Scatter plots showing the results of multidimensional scaling using beta diversity (Spearman correlation distance) calculated from metabolome profile. Plots were color-coded by subject (**E**) or time point (**F**).

We conducted a randomized, double-blind controlled trial of 19 Japanese subjects (Figure 1A,B). Baseline clinical characteristics were similar in primary and secondary outcomes, including blood glucose AUC and iAUC, insulin AUC and iAUC, fasting blood glucose and insulin and stool frequency in both groups (Tables S6 and S7). First, we statistically tested each primary and secondary outcome; however, there were no significant differences observed in any of the outcomes (Table 1). Effects for other blood parameters are written in Table S8.

### 3.2. Effect of Barley Intake on Intestinal Microbiome and Metabolome Profiles

To evaluate the effect of barley on intestinal microbiome and metabolome profiles, we performed 16S rRNA gene-based microbiome analysis and CE-TOFMS-based metabolome analysis. Multidimensional scaling with beta diversity showed that the plots from each subject were clustered in both microbiome and metabolome profiles (Figure 1C–F and Figure S1). Comparison of inter-individual distances and intra-individual distances showed significant differences (Permutational multivariate analysis of variance (PERMANOVA) test, *p* = 0.001 in both microbiome and metabolome profile), suggesting that the individual difference was larger than the influence of barley or control food consumption.

**Figure 2:**
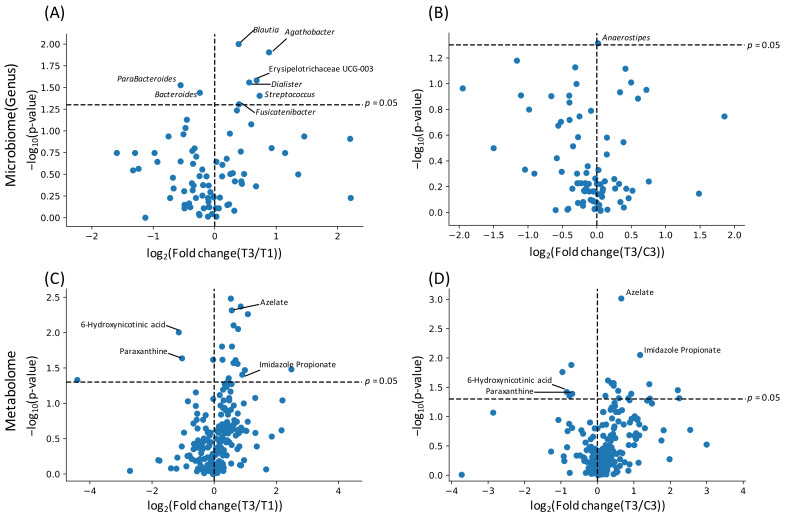
Effect of barley intake on intestinal microbiome and metabolome. *X* axis indicates log fold change in mean value of corresponding genus/metabolite abundance after 4-week intervention relative to control time point. *Y* axis indicates logarithmic value of *p*-value. Each bacterium (**A**,**B**) and metabolite (**C**,**D**) were plotted. T1 (**A**,**C**) and C3 (**B**,**D**) were used as control time points.

We next performed the Wilcoxon signed-rank test for the relative abundance of each microbe and the relative area of each metabolite, to further investigate the effect of barley consumption on the intestinal environment. The comparison was performed between pairs of two different sets; T1–T3 and C3–T3 (Figure 2). Some genera showed significant differences in their relative abundance (*p* < 0.05, not corrected), such as *Blautia* and those belonging to the family Lachnospiraceae in T1–T3 comparison; however, these differences were only observed as significant without multiple testing correction (Figure 2A,B; Table S9). Several metabolites showed significant increases (e.g., azelate and imidazole propionate) and decreases (e.g., paraxanthine and 6-hydroxynicotinic acid) in their relative area; however, the significance was only observed without multiple testing correction (Figure 2C,D; Table S10).

### 3.3. Characteristics of Barley Responders with Glucose Tolerance Improvement

In a previous study, subjects were classified into responder and non-responder groups, based on phenotypic criteria, to investigate the intestinal characteristics of subjects who showed improvement in glucose tolerance due to barley consumption [6]. We also adopted the criteria of glucose tolerance responders, reported in the study of Kovatcheva et al., revealing that only two or three subjects (subject 14 and 23 at T2, and subject 01, 02, and 06 at T3) were defined as glucose tolerance responders. Since analysis with a small sample size could lead to incorrect results, we did not perform stratified analysis and instead performed correlation analysis. Specifically, for each subject, we defined the degree of glucose tolerance index improvement (referred to as glucose tolerance responder score), and performed correlation analysis between glucose tolerance responder score, and either baseline feature or change of feature by barley intake.

**Figure 3:**
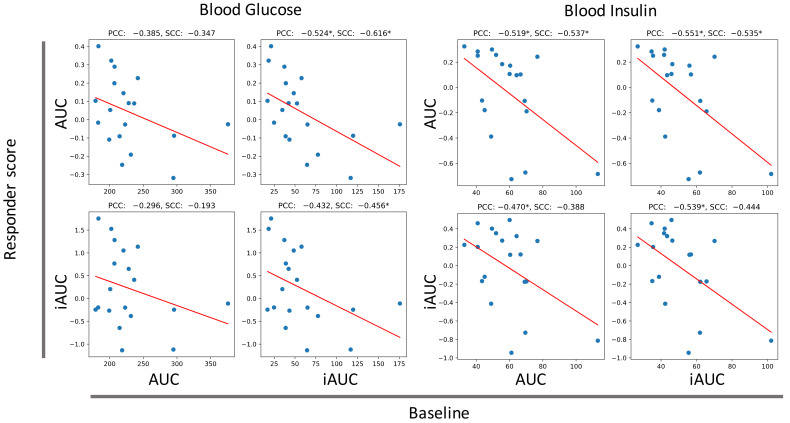
Improvement of glucose tolerance score by barley intake depends on glucose tolerance score at baseline. *X* axis indicates glucose tolerance index at baseline and *Y* axis indicates responder score. PCC: Pearson correlation coefficient, SCC: Spearman correlation coefficient, * *p* < 0.05, no correlation test.

First, we analyzed glucose tolerance responder score and glucose tolerance index at baseline. We used blood glucose AUC/iAUC and insulin AUC/iAUC as the glucose tolerance indices. As a result, blood glucose AUC/iAUC and insulin AUC/iAUC improved in the individuals with high blood glucose AUC/iAUC and insulin AUC/iAUC at baseline, respectively (Figure 3). Interestingly, the improvement of blood glucose AUC/iAUC does not depend on insulin AUC/iAUC at baseline, and vice versa (Figure S2). This suggests that barley regulates glucose tolerance towards a healthier state and the improvement of the levels of blood glucose and insulin are independent of each other.

**Figure 4:**
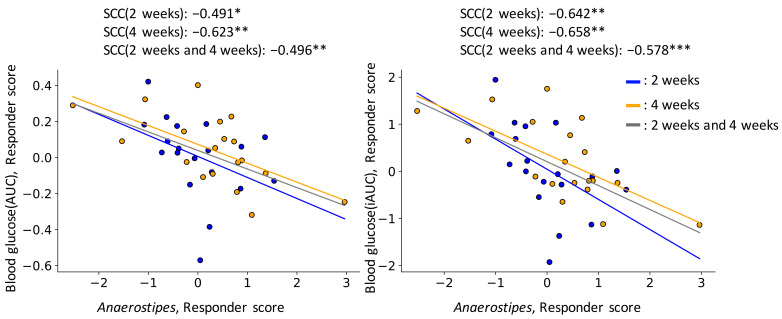
Increasing of *Anaerostipes* correlates with improved glucose tolerance. *X* axis indicates *Anaerostipes* responder score, and *Y* axis indicates blood glucose AUC/iAUC responder score. SCC indicates Spearman correlation coefficient and asterisks (*) indicate statistically significant correlated (no correlation test). *, *p* < 0.05; **, *p* < 0.01; ***, *p* < 0.001.

Subsequently, we analyzed intestinal environmental changes that correlated with glucose tolerance index improvement by barley intake. As improvement of blood glucose AUC/iAUC did not depend on insulin AUC/iAUC at baseline, improvement analysis was performed independently. Although there were hundreds of intestinal bacteria and metabolites, few of them consistently correlated with blood glucose AUC/iAUC or insulin AUC/iAUC responder scores across two time points (intake 2 weeks and 4 weeks). Only the increase of *Anaerostipes* by barley intake was consistently correlated with blood glucose AUC/iAUC improvement across two time points (Figure 4; Table S11). Remarkably, the *Prevotella*/*Bacteroides* ratio was not correlated with glucose tolerance index improvement, although the *Prevotella*/*Bacteroides* ratio was previously reported to increase after barley consumption in responders [6]. Blood glucose AUC/iAUC improvement correlated with some genera of *Prevotella* (*Prevotella* 2 and *Prevotella* 7) only at T3.

## 4. Discussion

In this study, the effect of barley on glucose tolerance was quantified. In addition, by conducting a comprehensive analysis of intestinal microbiome and intestinal metabolome, we quantified the effects of barley on the intestinal microbiome and metabolome. Furthermore, we found the relationship between barley’s improvement in glucose tolerance and changes in the intestinal microbiome and metabolome.

In the microbiome, microbial genera that belong to the family Lachnospiraceae, such as *Blautia*, *Agathobacter*, and *Fusicatenibacter*, showed significant increase in their relative abundance after barley consumption, compared to baseline (Figure 2A). Among those genera, *Blautia* and *Agathobacter* were previously shown to be increased by the intake of whole grain barley [16]. We used pearled barley (which lacks the bran and germ, and mainly consists of endosperm) as the test food in our study. The endosperm and its contents contained in whole grain and pearled barley may be important factors for increasing these bacterial abundances. β-glucan is a soluble dietary fiber and a major component of endosperm, suggesting a strong effect of these fibers for the gut microbiota. It has been reported that *Blautia* abundance and visceral fat area is negatively correlated [17], and it is less prominent in the intestines of patients with diabetes or cirrhosis [18,19]. As such, *Blautia* is thought to be a putative biological marker for diseases related to metabolic syndrome, such as obesity or diabetes. Although it is yet to be determined whether the increase in the abundance of *Blautia* has beneficial effects on these diseases, it could lead to improvement of their symptoms or conditions. *Agathobacter* is a bacterial genus where Eubacterium rectale has been reclassified and has been reported to be a dominant producer of butyric acid. In addition, *Agathobacter* is known to be less prominent in the intestines of patients with ulcerative colitis, as compared to those of healthy individuals [20]; however, a significant increase of butyric acid was not detected in this study. In a previous study, it was suggested that various subspecies exist within the *Agathobacter* genus, and subspecies that are dominant vary depending on the country [21]. There is a possibility that the *Agathobacter* subspecies dominant in Japanese subjects’ intestines do not produce butyric acid, and it is also likely that although intestinal levels of butyric acid increase, fecal levels of butyric acid remain unchanged due to the produced butyric acid being absorbed in the small intestine [22].

In the metabolome profile, significant differences were observed in levels of azelate, paraxanthine, 6-hydroxynicotinic acid, and imidazole propionate in T3, compared to T1 and C3 (Figure 2C,D). As azelate is a common nutrient found in grains, including barley, it may not be increased due to the metabolism of intestinal microbiota. It has been reported that the administration of azelate to mice improves glucose tolerance, suggesting that it may be a factor contributing to glucose tolerance improvement by barley consumption [23]. A previous report has shown that imidazole propionate impairs insulin signaling via activating mechanistic targets of rapamycin complex 1 [24]. If emission of imidazole propionate from intestine is increased, a beneficial effect is assumed, but if intestinal imidazole propionate production is increased, it may lead to the deterioration of glucose tolerance. In addition, even in high dietary fiber-containing barley, which is the substrate for short-chain fatty acid (SCFA) production, we did not detect any significant differences in SCFA levels at any time point.

During the study, subjects who showed improvement in the barley consumption-induced glucose tolerance had significantly high blood glucose iAUC prior to the barley intervention, suggesting that barley moderated the glucose tolerance towards a healthier state. Subsequently, we explored bacteria and metabolites that correlated with blood glucose AUC/iAUC and insulin AUC/iAUC responder scores. A previous study showed that barley consumption increases the *Prevotella*/*Bacteroides* ratio in barley-consuming healthy Swedish subjects with improved glucose tolerance [6]. In this study, the increase of *Prevotella*/*Bacteroides* ratio is not correlated with blood AUC/iAUC improvement but *Prevotella* 2 and *Prevotella* 7 are correlated with blood glucose AUC/iAUC improvement. It is assumed that i) the increase of *Prevotella*/*Bacteroides* ratio by intake of barley is not maintained for a long time (intervention period was 3 days in the previous study and 14 days and 28 days in this study) ii) functions are different between various strains of Prevotella in multiple countries [8]. *Anaerostipes* was correlated with blood glucose AUC/iAUC improvement consistently, and abundance of *Anaerostipes* was higher in the low glycemic index diet intake group [25]. In addition, in two independent studies, it was found that the presence of *Anaerostipes hadrus* that encodes a composite inositol catabolism-butyrate biosynthesis pathway resulted in lower host metabolic disease risk [26]. Since *Anaerostipes* spp. are known as a butyrate-producing bacterium [27], and butyrate improves glucose tolerance through several mechanisms, such as gut-brain axis [28] and induction of GLP-1 [29], barley consumption assumably results in an increase in *Anaerostipes* spp., followed by butyrate production. However, in this trial, the fecal amount of butyrate was not correlated with the improvement in glucose tolerance. This may be due to the reason that the amount of butyrate in the feces does not necessarily reflect the local amount of butyrate produced by *Anaerostipes* spp. in the intestine because butyrate is absorbed quickly and/or used as energy source in the intestine. In addition, a previous study described that there are structural variations in the *Anaerostipes hadrus* genome. In 16S rRNA gene analysis, it is unclear whether *Anaerostipes* spp. encodes composite inositol catabolism-butyrate biosynthesis pathway gene [26], so additional testing using shotgun metagenomics may be necessary.

This study has a few limitations. First, the amount of some metabolites in the feces may not always reflect the amount in the intestine, as some metabolites were absorbed in the intestine. Second, the control food itself also contained dietary fibers. Dietary fiber acts as a prebiotic, also known as microbiota-accessible carbohydrate (MAC), to support the production of SCFAs by gut microbiota [30], thus having potentially similar effects as barley. Therefore, it may be difficult to see the impact of test food clearly, as compared with control food intake. Third, intestinal microbes and metabolites are complex parameters which contain many different bacterial genera and metabolites. FDR correction is necessary in statistical hypothetical tests; however, as many items were comprehensively observed, the FDR correction was strict, increasing the difficulty of detecting the true significant differences. Considering, in addition, that the sample size in this study was not large (19 subjects), validation tests using models such as murine models should be considered to confirm these findings.

## 5. Conclusions

This randomized controlled study provided novel insights into the effect of barley on the intestinal environment and blood parameters in a Japanese population. Barley intake increased some intestinal bacteria such as *Blautia* and *Agathobacter*, and metabolites such as azelate. In addition, the improvement of glucose tolerance due to the effect of barley intake was dependent on the baseline glucose tolerance. Further, increasing the gut *Anaerostipes* is possibly involved in its improvement. In this study, we integrated analysis of the intestinal microbiome and metabolome and analyzed the relationship with improvement of glucose tolerance, uncovered the impact of diet on the intestinal environment, and shed light on stratified health care considering the intestinal environment.

## Figures and Tables

**Table 1:** Effect size and *p*-values of primary and secondary outcomes.

	C2 vs. T2		C3 vs. T3	
	Effect Size (95% CI) *^1^	*p*-Value *^2^	Effect Size (95% CI) *^1^	*p*-Value *^2^
Blood glucose AUC (0–120 min)	−0.019 (−0.461 to 0.424)	0.934	0.268 (−0.180 to 0.723)	0.238
Insulin AUC (0–120 min)	0.018 (−0.425 to 0.460)	0.937	−0.123 (−0.569 to 0.320)	0.582
Blood glucose iAUC (0–120 min)	−0.099 (−0.544 to 0.343)	0.657	0.258 (−0.189 to 0.712)	0.256
Insulin iAUC (0–120 min)	0.018 (−0.425 to 0.460)	0.937	−0.091 (−0.536 to 0.351)	0.682
Fasting blood glucose	0.241 (−0.205 to 0.694)	0.287	0.021 (−0.421 to 0.464)	0.924
Fasting blood insulin	0.009 (−0.434 to 0.451)	0.968	−0.277 (−0.733 to 0.171)	0.223
Stool frequency	0.122 (−0.321 to 0.568)	0.585	0.267 (−0.181 to 0.722)	0.240

*^1^ Effect size (paired Hedge’s g) and 95% confidence interval (CI). *^2^ *p*-value was calculated by paired *t*-test.

## Data Availability

All 16S rRNA amplicon sequence files generated in this study are available in DRA of DDBJ (DRA accession number: DRA009319).

## Supplementary Materials

The following supporting information can be downloaded at: https://www.mdpi.com/article/10.3390/nu14173468/s1, Table S1: CONSORT checklist; Table S2: Key inclusion/exclusion criteria; Table S3: Relative area of metabolome in feces; Table S4: Amount of metabolome in feces; Table S5: Microbiome phylogenetic composition; Table S6: Group, gender, age and BMI in the study; Table S7: Difference in base stats and primary/secondary outcome in baseline; Table S8: Difference in blood parameter between timepoints; Table S9: Difference in gut microbiota between timepoints; Table S10: Difference in gut metabolite between timepoints; Table S11: Significantly correlated bacteria and metabolites with blood glucose responder score. Figure S1: Individual differences were larger than the influence of barley or control food consumption on microbiome (weighted UniFrac distance); Figure S2: Improvement of blood glucose AUC/iAUC by barley intake does not depends on blood insulin AUC/iAUC at baseline and vice versa.
