# Supplementary material for: Metabologenomic Approach Reveals Intestinal Environmental Features Associated with Barley-Induced Glucose Tolerance Improvements in Japanese: A Randomized Controlled Trial
Source: Nutrients. 2022 Aug 24;14(17):3468. doi: 10.3390/nu14173468 (PMC9460218; doi:10.3390/nu14173468)
Supplement: Supplementary file 1 [file nutrients-14-03468-s001.zip › nutrients-1853911-Supplementary Figures.pdf]

Supplementary Figures

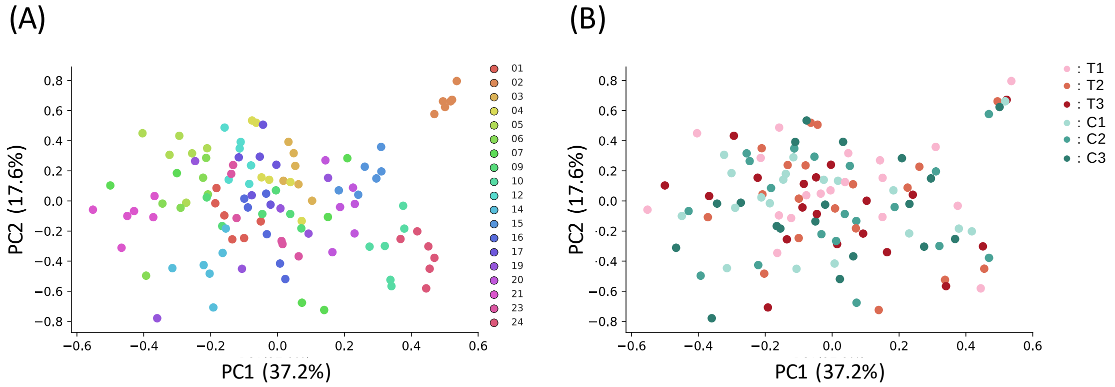

Figure S1. Individual differences were larger than the influence of barley or control food consumption on microbiome (weighted UniFrac distance)

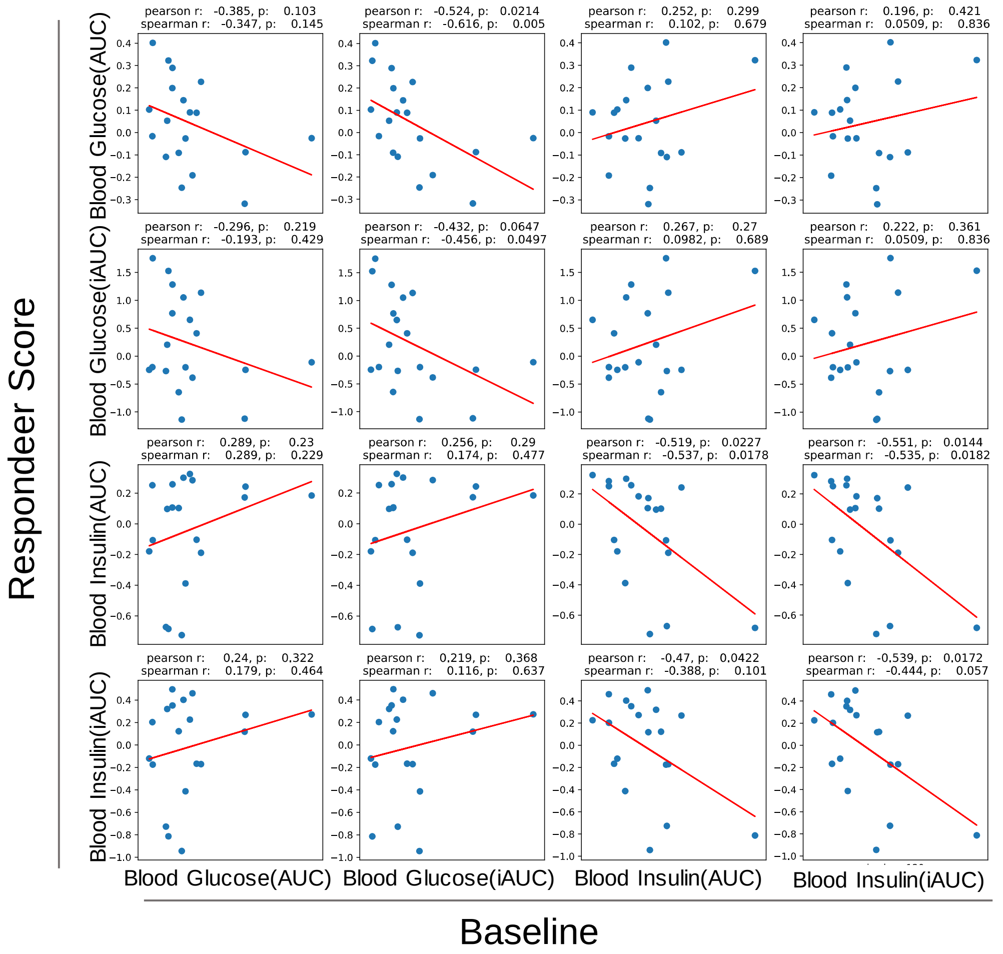

Figure S2. Improvement of blood glucose AUC/iAUC by barley intake does not depends on blood insulin AUC/iAUC at baseline and vice versa.
